# Dr. Patterson, on New Discoveries

**Published:** 1801-11

**Authors:** W. Patterson

**Affiliations:** Londonderry


### To Dr. BRADLEY.

Dear Sir,

As your Journal is not only medical but phyfical, the following *Sketch* feems to come within its appointed fcope, and, therefore, I addrefs it to you for infertion, provided you think that it will not be unworthy of its room. I am not infenfible to the value of honeft fame to an individual, nor blind to the advantages arifing to the public from his defire of repute, neither am I difpofed to juftify an envious carping at inventive genius; but I am folicitous to encourage modeft merit, and to hinder unaffuming man, of native abilities, from being daunted with the din of trumpeted difcoveries. Hence it is my intention, in the fubfequent animadverfions, to inveftigate generally the real merits of thofe, called inventors, or original difcoverers, and to try the juftice of their title to thefe honorable appellations. As in the cafe of a nation, fo in that of a fingle perfon, it is at once flavifh and detrimental to fubmit to the univerfal influence on the opinions of mankind, which they, who conceive themfelves to be creative geniufes, are prone to aim at, by holding forth their works as inftances of novelty, or models of perfection, undefcried or unequalled at any former time by any other of the human race.

The labours of an extraordinary genius, it is true, in eftablifhing the objects of his purfuit, are apt to dazzle us with their fplendour, or awe us with their profundity, and induce us to concur in the fancied boundary of human knowledge. But who can pretend to fay, that thus far the human intellect fhall go, and no further? Who will venture to affert, that here fhall the proud waves of the intellectual powers be ftayed, and vainly beat againft an infurmountable barrier? Who would with weary fteps travel over the beaten path, to places already known, if he had not in profpect the undifcovered country, to urge on his hope and ambition? The man of talents and induftry, I am certain, may yet continue to apply all his faculties with unabated vigour, and unapalled expectation of advancing in the profecution of his defigns. It is a vifionary idea to fuppofe, that human degeneration will bring on a bankruptcy of the literary world fooner than the laft univerfal wreck! The poffible exiftence of fuch a world of retrograde beings, is no more deplorable than ridiculous, wandering on, as it were, ufelefs and exhaufted, and bewailing their condition. Convinced of the inexhauftible fources of human capacity, I venture to affirm, that in all fimilar ftages of fociety, ftamping on the the ground would raiſe up a genius, perhaps a hoſt of geniuſes, equal to any that had gone before in ſuch walks! Wiſhing then, that the ample plain, which Nature has provided for the mind of man, be left open for its uncontrolled and undaunted courſe, I beg your acceptance of

### A SKETCH,

### Concerning the Queſtion,

### Is there any thing which can with juſtice be pronounced *Entirely New?*

At a remote period in civilized ſociety, nearly 2,800 years ago, a wiſe and eloquent preacher aſked, 'Is there *any* thing whereof it may be ſaid, ſee, this is *new?*' To which queſtion an anſwer is furniſhed by the ſame ſagacious obſerver, who pronounces, 'It hath been already of *old time*, which was *before us*.'* In examining the progreſs of ſociety, we ſhall find that it has certain ſtages, and that men, in parallel ſituations, muſt think and act alike. The circle of human events is not immenſe; and in its revolution it muſt happen, that the ſame point in the circumference will, repeatedly, be uppermoſt. 'The wind goeth toward the ſouth, and turneth about unto the north; it whirleth about continually, and the wind returneth again according to his circuits.'† That appearance of novelty, which the face of things wears to the bulk of mankind, is nothing but a *ſhow*; what men act, men have acted; what men think, men have thought. Yet, in this round, whilſt numerous and ſtriking inſtances of great attainments in ſcience and arts are obſerved, man does not ceaſe to be the man of Nature.

It is true, that in paſſing through the different periods of the ſocial ſtate, man ſucceſſively acquires the ideas which, in their turns, thoſe periods are fitted to ſupply; but the gradual improvements which he makes in that ſtate, are only thoſe which Nature permits, or even which ſhe commands him to make at the ſtages which ſhe herſelf has fixed for his exertions. To the ſocial progreſs, at the different ages of ſociety, belong certain ſums of intellectual property, originally, and without borrowing from exterior funds. Thus, the ancient Peruvians, without the help of either the Egyptians, the Phœnicians, or the Greeks, had eſtabliſhed wiſe laws, and, in certain points, had made great advances in the arts and ſciences.

In Aſia can be traced evident veſtiges of governments of

* Eccleſiaſtes, chap. I. v. 10.

† Ibid. v. 6.

extreme antiquity, and far advanced in the fublimeft fciences, now dwindled into new and inferior forms; *there* likewife may be difcerned, in the living manners and fentiments, ideas which Europeans have contemplated with wonder and admiration, in both facred and profane writings; and *there* may be obferved many things conducive to the purpofes of life, unknown to the refined nations of Europe, preferved during thoufands of years, amidft many revolutions, which, though they change the families of the fovereign, pay greater refpect to eftablifhed laws, cuftoms, and modes of thinking, than the more fweeping convulfions of our weftern nations.

In Afia, alfo, many operations, which in Europe cannot be performed without a complication of machinery, are executed with much greater fimplicity and eafe. In that primitive and moft effential art, *agriculture*, wherein England has been fuppofed to furpafs all the reft of the world, Afia, it appears, is greatly her fuperior on the fcore of antiquity, and her competitor, at leaft, in fome important branches of the employment. The model of a plough, for inftance, ufed in the drill hufbandry, has been lately brought from India, which, for fimplicity of conftruction, eafe of management, and lownefs of price, is efteemed vaftly preferable to any thing of the kind that has been hitherto known in thefe European countries.

It is held a queftion, if there exift in Nature fuch a being as a man of real creative genius? No doubt, there are in the mental capacities very difcernible fhades; no doubt, the fciences and the arts, in their courfe from age to age, are enlarged and perfected; no doubt, difcoveries are made, and inventions proclaimed, but they are never on a fudden, and confequently, no one difcovery or invention, fo called, ought to be confidered as the work of an individual. That which we name *invention*, therefore, ought, in reality, to be deemed only a fucceffion of trials and attempts which follow each other, more or l fs eafily or laborioufly, in a feries of ages. Hence it may be inferred, that the men to whofe names it is cuftomary to attach the whole celebrity, would, in feveral cafes, find it difficult to recognife all the parts of the work imputed to them, or even to comprehend the leffons of thofe who believe themfelves, and above all, nominate themfelves, their difciples.

Several branches of knowled e, which have been fuppofed, or claimed, to be of modern production, were certainly known to the ancients, as facts at leaft, if they were unacquainted with the true methods of explaining the caufes. The Chinefe were acquainted with the ufe of the mariner's compafs, with the compofition of gunpowder and porcelain, and with the art of printing long before the Europeans. Dr. Falconer, in the

Manchefter

Manchester Memoirs, plainly shows, that what was reckoned an original discovery of Dr. Black, viz. 'that water which had been boiled, was more easily frozen than water that had not undergone that operation,' was known to Hippocrates, Aristotle, Pliny, Galen, and others. He also brings several passages from ancient writers to prove, that Dr. Black's doctrine of latent heat was well known to them. Juvenal, the Roman satirist, who lived in the same century (first) with Pliny, observes, that water is rendered more susceptible of coagulation by boiling it:

'*Frigidior Geticis petitur decocta pruinis.*'

In the East Indies, where ice is prepared by means of evaporation, it is found necessary to boil the water, to facilitate and perfect the process, a piece of knowledge, which, it does not appear to us, that the Easterns received from Dr. Black, or from any other European.

It has been lately shown, that the discoveries in chemical philosophy and physiology, which have been ascribed to Mayow, relative to the composition of the atmosphere, its nitro-aerial quality, and its use in respiration, had been anticipated and published by Dr. Hook, from four to ten years earlier than Mayow; the Micrographia of the former having been printed in 166[illegible], whilst the Treatise of the latter, on Respiration, was not given to the public until 1668, and his Treatise on Nitre, &c. not until 1674.*

Perhaps all the authors, to whom great discoveries in philosophy have been ascribed, have, in truth, rarely or never ascended far above the common level of the philosophical knowledge of the ages in which they respectively lived. The harangues and discussions in the public assemblies at Athens, the moral knowledge which the people had generally acquired, the writings and exhibitions of the drama, the errors and attainments of former philosophers, and a variety of other circumstances, had so prepared the way for the entry of the philosophy of Socrates, as to make it impossible that there [illegible]ould not some such philosopher arise amongst the Athenians about that æra. Aristotle was but a disciple of the Socratic school, from the doctrines of which, and from those of the cotemporary sophists, his logics and scientific arrangements derived their birth.

The discoveries assigned to Bacon, celebrated as being scientific by *instinct*, were made at a time when the world began to grow weary of the logic and metaphysics of the schools; and when

* Philosophical Magazine, No. XII. p. 370.

when experiment and induction had been already tried with fuccefs; and when a fortunate coincidence of other caufes confpired to favour his genius for fcientific contemplation. The refearches of Galileo, if they did not difcover the gravity of the atmofphere, yet they approached fo near it, as to leave no very extraordinary merit to his pupil Torricelli in its actual accomplifhment. And if we dared, with an unawed eye of impartial revifion, to approach the hallowed monumental tablet of the great Newton's fame, we might difcern, that the meed of renown ought, in reality, to be divided betwixt him and other inquirers, amongft whom Galileo and Kepler would deferve a confiderable portion.*

It is remarkable, that fome of Sir Ifaac Newton's moft refined fpeculations were neither peculiar to himfelf, nor new to other people, not even to thofe nations whom we hitherto have been taught to confider dull and illiterate. The whole of his theology, and part of his philofophy, are found in Afiatic writings. That *most subtile spirit*, which he fufpected to pervade natural bodies, and, lying concealed in them, to caufe attraction, repulfion, refraction of light, electricity, &c. is defcribed by the Hindus as a *fifth element* endued with thofe very powers. †

Becaufe Newton made confiderable progrefs in the theory of light, difcoveries in this fubject, which really belong to others, have been afcribed to him. For inftance, he is faid to have difcovered, that the refrangibility of light is in the inverfe ratio of that of all other bodies; whereas this he did not difcover. Defcartes firft publifhed the difcovery of the conftant ratio of the feries of incidence and refraction, though it was certainly known before to Willebrord Snellius; and Kepler was very near difcovering it. ‡ But, if Newton's doctrine of light and colours be fundamentally erroneous, as lately has been alleged, his followers will of courfe no longer contend for his right to the honour of difcovery. From a careful repetition of the experiments by which Sir Ifaac effected his analyfis of light, phenomena, materially different from thofe which appeared to him, were difcovered, and found to lead to a change of his theory of light and colours. This antagonift to the Newtonian hypothefis concludes from the whole, that Sir Ifaac has erred in believing every ray of common light to be compofed of feven differently coloured, primogenial, elementary rays. On the contrary,

* Monthly Magazine, Vol. V. p. 117.

† Afiatic Refearches, Vol. IV.

‡ Thomfon's Fourcroy, Vol. I. p. 140.

contrary, this author's obfervations enable him to infer, that all light is originally of one uniform *white colour*; that its diverfity of colours, in inflexion, is occafioned by the bendings, feparations, and other changes of its parts in paffing through a tranfparent medium, or under attraction by the edge, angle, or fide of an approaching body; and that, by confequence, the Newtonian theory of light and colours is not fundamentally true. This oppofite theory has a juft claim to perfect credence, if accuracy of obfervation, logical fairnefs of induction, and a good ftyle of compofition, can eftablifh its titles. The fame doctrine has been more haftily and obfcurely fuggefted by Mr. Heron, in his Elements of Chemiftry.*

With refpect to that moft important difcovery, the *circulation of the blood* in the human body, inftead of the immortal Harvey, as he is ftyled, having a right to the entire merit of it, we fhall find on a cautious review of its hiftory, that the way was paved by other anatomifts and phyfiologifts, and that he did little more than corroborate their obfervations. It is highly probable that Harvey obtained the foundation of his knowledge in Italy, where he ftudied with remarkable diligence under the famous anatomift, Fabricius ab Aquapendente, who certainly was mafter of the doctrines of Vefalius, Servetus, Columbus, and Cæfalpinus, whofe labours had almoft compleated this field of inveftigation. It was in the very end of the 16th century that Harvey, as was the cuftom of the times, went to ftudy medicine in Italy, and muft have learned the exiftence of the valves of the veins from Fabricius, who had difcovered them fome years before, and doubtlefs demonftrated them in his lectures. The great Vefalius, near 100 years before Harvey, publifhed a noble fyftem of anatomy. Servetus, followed by Columbus, both in the times of Vefalius, had clearly given the circulation of the blood in the lungs, which Dr. Hunter reckons, at leaft, three quarters of the difcovery; and Cæfalpinus had many years before Harvey, publifhed, in three different works, all that was wanting to make the doctrine of the circulation complete. From the difcoveries refpecting the veffels, and courfe of the blood through the lungs; from the obvious phenomena of ligatures on different veffels; from the practice of furgery, with regard to bleeding and blood-veffels; from the action of the heart when expofed to view in living bodies; from all thefe circumftances, which fo evidently proclaim the circulation, nothing more feems to have been required for crowning the difcovery,

* Philofophical Magazine, No. XXVIII. p. 365.

covery, than laying afide grofs prejudices, and fairly confider-ing obvious truths.

In merit, therefore, Harvey's rank muft be comparatively low as a difcoverer. So much had been found out by others, that little more was left for him to do, than to drefs it up into a fyftem; and that art, every judge in fuch matters will allow, required no extraordinary talents. Yet, eafy as the undertaking was, it procured for him the epithet, *immortal*. "But none of his writings," fays Dr. Hunter, "fhew him to have been a man of uncommon abilities. It were eafy to quote many paffages, which bring him nearly to a level with the reft of mankind. He lived almoft thirty years after Afellius publifhed the Lacteals; yet, to the laft, feemed moft inclined to think, that no fuch veffels exifted. Thirty hours, at any time, fhould have been fufficient to remove all his doubts." And what leffens ftill more Harvey's claim to diftinction is, that fo far from his doctrine being perfect and well founded, with the ancients he fuppofes, in contradiction to an actual law of the circulation, that the blood moves from the heart through the veins. He either knew not, or did not attend to that effential part of the fyftem, the contractile power of the arteries; and he did not admit, or at leaft very indiftinctly conceived, the immediate communication of the arteries and veins.*

Similar ftrictures, perhaps, might with juftice be made on fome of our cotemporaries, who affume the exclufive credit of difcovering the abforbent fyftem. Look at what was done, fince the year 1627, by Afellius, Pecquet, Rudbec, Bartholine, Highmore, &c. concerning the lacteals and lymphatics, and then remark what was left for recent labourers in this field, or how they could fail in bringing it to the prefent ftate of culture.

Adverting to another effential function of the human fyftem, namely, the act of *Refpiration*, the merit of difcovery concerning its laws is very much divided. On this fubject, the opinions of the ancients were merely conjectural; and notwithftanding many ingenious attempts in after ages, no fatisfactory doctrine appeared till the year 1776, when that profound chemift and much lamented martyr, Lavoifier, publifhed an hypothefis, which laid the foundation for the fubfequent advances in thi walk. To the labours of Lavoifier were added thofe of Cigna, Prieftley, Crawford, &c.; the latter of whom unfolded one

* Flemyng's Lectures, Lect. IV. Aikin's Memoirs of Medicine, p. 288, et feq. Hunter's Introductory Lectures, p. 40—47.

one of the moſt important uſes of reſpiration, that is, its office in deriving the element of animal heat from the air. But this beautiful theory of reſpiration is yet attended with ſome difficulties, which we hope ſoon to ſee entirely overcome by the ingenuity, induſtry, and zeal of modern enquirers.

In the inſtance of what is called the new and true theory of *Chemiſtry*, does the merit of diſcovery belong to Lavoiſier? Or rather, are we not to ſhare the honour betwixt Van Helmont, Boyle, Black, Macbride, Prieſtley, &c. who had, ſucceſſively or collaterally, purſued chemical inveſtigations, and traced out the general truths of this ſcience, until it was almoſt impoſſible that ſome one or other ſhould not ſtumble on the diſcoveries of the French philoſopher. In fact, were we minutely acquainted with the ſteps by which claimants of grand diſcoveries were conducted to their knowledge, we ſhould not fail to abate much from the fervour of admiration and applauſe which we at preſent beſtow on thoſe perſons. Yet in philoſophical, as in other caſes, we are at all times too apt to praiſe him who impoſes the key-ſtone, as if he had built the whole arch.*

Many more examples might be adduced to illuſtrate and confirm the truth of the general poſition, that in reality there is not any thing under the ſun whereof it can be ſaid, "*This is abſolutely new*," but more would be ſuperfluous, and would be inconſiſtent with the nature of a *ſketch*. I cannot, however, omit this opportunity of teſtifying the gratification which I enjoy from obſerving, in the courſe of this ſurvey, the diſtinguiſhed rank to which my countrymen are juſtly entitled in the republic of letters. The names of Boyle, Eeles, Macbride, Kirwan, and Crawford, I obſerve engraved high on the column of ſcience, and ſhould remain for ever engraved on the hearts of the friends of our united kingdom, of philoſophy, and of mankind. Nor can I omit this opportunity of doing juſtice to the memory and merits of one of theſe deſerving men, the late ingenious and amiable Doctor Macbride. In the courſe of a ſet of experiments made to aſcertain the ſolvent power of quicklime, and firſt publiſhed in the year 1764, Dr. Macbride diſcovered, that an artificial ſulphureous water could be prepared, reſembling the natural kind in all its ſenſible properties; and, in his apparatus on this occaſion, he employed an *air-valve*, ſuggeſted to him by an ingenious friend, Wm. Deane, Eſq. and which has been introduced into ſucceeding machines for a ſimilar purpoſe.

* Monthly Magazine, Vol. V. p. 117.

Many years before that time, it is true, Boyle, Hales, and others, made numerous experiments on air, and chiefly on the analytical plan too, but not with a deſign of decompounding the conſtituent elements, ſo as to reproduce the original ſubſtances. Later experimenters did little more. Dr. Siep, of Pyrmont, in the year 1736, and Dr. Brownrigg, of Whitehaven, in the year 1765, communicated to the Royal Society of London, their ſuggeſtions relative to the reſidence of carbonic acid gas, or fixed air, in natural Spaw waters; but, although they decompoſed theſe waters, they do not furniſh us with any expedients for imitating them by means of art. Dr. Falconer alſo publiſhed a Treatiſe on the Bath Waters in the year 1772, wherein he treats of carbonic acid gas, as being an ingredient in many natural ſpaws, but drops no hint, in that edition, about compoſing ſimilar waters, by imparting the gas to common water. Nor does Dr. Rutherford, who, at the latter end of the ſame year, wrote an ingenious diſſertation on carbonic acid gas, give the ſmalleſt intimation of his being acquainted with any method of making factitious mineral waters.

In that very year, however, viz. 1772, Dr. Prieſtley came forward, contending in a famous pamphlet for the honour of a diſcoverer, and denying the title of every other perſon to even a ſprig of laurel on the occaſion. But, as to the validity of this claim, it need only be remarked, that Dr. Macbride's book was before the public, and very generally read and applauded, *eight years* antecedent to the appearance of Dr. Prieſtley's pamphlet. Dr. Nooth, to whom the firſt deſign of the *Glass Machine* is aſcribed, whilſt admitting that Dr. Prieſtley was the only perſon who had *published* any deſcription of an apparatus purpoſely for communicating carbonic acid gas to aqueous fluids, aſſerts that various methods for doing ſo were contrived, at fartheſt back, from the year 1766, but were not publickly notified at the time; yet he makes no mention of Dr. Macbride's propoſal, though it was given to the world two years prior to that date. As to Mr. Venel's extract from "*Memoires de Mathematique et de Physique*," it did not appear until after Dr. Prieſtley's pamphlet had been tranſlated into French; and was not intended to vindicate to himſelf Dr. Prieſtley's ſuppoſed diſcovery, but in reality to claim precedence of Dr. Brownrigg. So that from this ſtate of facts, we may juſtly conclude, that Dr. Macbride, aſſiſted in one point by his countryman, Mr. Deane, is entitled to the credit of firſt ſuggeſting in theſe countries, not only factitious mineral waters, but alſo the baſis of that machine by which they now are ſo excellently compoſed. For, what Bacon ſays on this ſubject, in his work, "*De Dignitate et Augmentis Scientiarum, Lib. IV.*" are but looſe hints, or rather a kind of predictions, predictions, fuch as he throws out on many other points relating to arts and fciences.

Upon the whole, then, we may conclude in the words of the writer, whom we quoted at the beginning, "The thing that hath been, it is that which fhall be; and that which is done, is that which fhall be done: and *there is no new thing under the sun.*"

*Londonderry, Sept.* 20, 1801. W. PATTERSON.

P. S. It may not be amifs on this occafion briefly to note, that after Grew, Camerarius, &c. Vaillant by his Botanicon Parifienfe in 1718, paved the way for the Sexual Syftem of Botany by Linné, in 1735; and that Dr. Darwin's charming Englifh Poem on the Loves of the Plants, was preceded by the *Connubia Florum*, an elegant Latin poetical effufion, publifhed by Dr. de la Croix in 1728. The Poem in Vaillant is figned, Mac-Encroe, Hibernus, Medicinæ Doctor; and the two Epigrams are figned, Demetrius de la Croix, Doctor Medicus.

